# Harmful Use of Alcohol: A Shadow over Sub-Saharan Africa in Need of Workable Solutions

**DOI:** 10.3390/ijerph14040346

**Published:** 2017-03-27

**Authors:** Carina Ferreira-Borges, Charles D.H. Parry, Thomas F. Babor

**Affiliations:** 1 Global Health and Tropical Medicine, Instituto de Higiene e Medicina Tropical, Universidade NOVA de Lisboa, 1349-008 Lisboa, Portugal; 2 Alcohol, Tobacco and Other Drug Research Unit, South African Medical Research Council, Cape Town 7505, South Africa; Charles.Parry@mrc.ac.za; 3 Department of Psychiatry, Stellenbosch University, Cape Town 7602, South Africa; cparry@sun.ac.za; 4 Department of Community Medicine and Health Care, University of Connecticut School of Medicine, Farmington, CT 06030, USA; babor@uchc.edu

**Keywords:** alcohol consumption, sub-Saharan Africa, alcohol policies, alcohol industry, alcohol-related burden

## Abstract

Alcohol consumption and alcohol-attributable burden of disease in Africa are expected to rise in the near future, yet. increasing alcohol-related harm receives little attention from policymakers and from the population in general. Even where new legislation is proposed it is rarely enacted into law. Being at the center of social and cultural activities in many countries, alcohol’s negative role in society and contribution to countries’ burden of disease are rarely questioned. After the momentum created by the adoption in 2010 of the WHO Global Strategy and the WHO Regional Strategy (for Africa) to Reduce the Harmful Use of Alcohol, and the WHO Global Action Plan for the Prevention and Control of Non-Communicable Diseases, in 2013, little seems to have been done to address the increasing use of alcohol, its associated burden and the new challenges that derive from the growing influence of the alcohol industry in Africa. In this review, we argue that to have a positive impact on the health of African populations, action addressing specific features of alcohol policy in the continent is needed, namely focusing on particularities linked to alcohol availability, like unrecorded and illicit production, outlet licensing, the expansion of formal production, marketing initiatives and taxation policies.

## 1. Introduction

According to the World Bank, the total population of sub-Saharan Africa in 2014 was about 973 million people. Almost half (43%) of the people living in that region were under 14 years old [1]. With only about 30% of the adult population drinking alcohol, and with expected increases in the number of potential new alcohol consumers, especially young people and women [2], the African continent has been identified by the alcohol beverage industry and market researchers as a key area for alcohol market growth [3,4].

While the alcohol industry is expanding its commercial activities in Africa to increase sales, recent data points to the rising burden that this will impose in terms of morbidity and mortality in this continent [5]. Several studies have documented a change towards a “public, binge drinking culture over the weekends”, leading to important health, social and development consequences, and have underlined the need to protect the population in general, and women and children in particular, from alcohol-related harms [6,7,8,9]. Moreover, recent scientific publications and WHO reports on harmful use of alcohol in Africa have issued a “public health warning” in response to the large investments and aggressive policy and marketing activities of the alcohol industry in their strategy to capture new markets in this continent [10,11,12,13]. The merger of SABMiller and AB InBev, the worlds’ two largest beer producers, has been viewed as having far-reaching consequences for health in Africa [14].

After the momentum created by the adoption of the WHO Global Strategy to Reduce the Harmful Use of Alcohol in 2010, at the meeting of World Health Organization’s Regional Committee for Africa held in Equatorial Guinea in 2010 [15], and the WHO Global Action Plan for the Prevention and Control of Non-Communicable Diseases (NCDs) 2013–2020, in 2013 [16], little seems to have been done in most countries in the region to address the harmful use of alcohol, which is defined as a pattern of drinking that results in physical or mental health problems. In this review, we argue that alcohol policy responses in Africa need to be strengthened in order to prevent increased consumption among population, in particular youth and women. Furthermore, action needs to go far beyond whether an intervention merely works by addressing particular features of alcohol policy in the continent. This means focusing on particularities linked to availability, like unrecorded and illicit production, outlet licensing including unlicensed liquor outlets (shebeens), the massive increase in formal production (e.g., to replace home brews), new marketing initiatives and taxation policies. To better understand the current policy environment surrounding alcohol consumption, we begin by summarizing the alcohol-related burden in the African Region. We then describe the evolution of countries’ responses at the global and local levels, discussing actual policies in African countries, difficulties encountered and possible solutions. Finally, we highlight the risks likely to result from the growth in the influence of the alcohol industry in the continent. We conclude by emphasizing the need for more effective, tailored policies.

## 2. Epidemiology

The distribution of alcohol consumption across countries in Africa is very heterogeneous and the type of drinks consumed differ geographically. Around one third of all the alcohol consumed in Africa is “unrecorded” (1.8 L per capita, per year), often being home-brewed through artisanal production, either by fermenting malted grains, fruits, sugar cane, honey or palm trees or by distilling them [2]. Being at the center of social activity in many villages, towns and cities, and deeply embedded in cultural activities, its negative role in society and contribution to countries’ burden of disease is rarely questioned [17]. More women than men abstain from alcohol use, although the gap between men and women in heavy alcohol consumption also seems to be narrowing as a result of increased availability of alcohol and changes in the role of women in the society, as well as targeted marketing strategies. Specific gender-related adverse effects of alcohol such as fetal alcohol spectrum disorders that result from drinking alcohol during pregnancy need to be given special attention in the Region [13].

Alcohol per capita consumption (APC) in the WHO African Region accounted in 2010 for 6 L of pure alcohol, with the highest consumption levels situated in southern Africa, in countries like Namibia and South Africa. Low consumption levels can be found in the countries of North Africa and sub-Saharan Africa, like Niger, Senegal or Guinea [2]. The later represent large populations of adherents to the Islamic faith, which have very high rates of abstention. Total APC reflects both recorded and unrecorded consumption. For example, South African adult per capita alcohol consumption in 2010 equaled 11 L of pure alcohol. Of this consumption, 3% or 2.9 L per person, was estimated to be homemade or illegally produced alcohol, otherwise known as unrecorded alcohol [2]. The consumption of homemade or illegally produced alcohol may be associated with an increased risk of harm because of unknown and potentially dangerous impurities or contaminants in these beverages. Those of lower socio-economic status experience greater harm as they have less access to health-care resources and have more deprived living conditions [18].

APC figures in Africa require a more in-depth analysis. This analysis needs to be contextualized taking into consideration demographic characteristics, rates of abstinence and patterns of consumption, first because the total APC will not reflect future increases in the total alcohol consumed in the population due to Africa’s demographic characteristics (very young population). Secondly, an APC of 6 L in a context where the majority of people are abstainers (58% of the population) must be seen differently from a region where, for example, only 10% are abstainers. In fact, the average amount consumed per drinker in this region, nearer to about 20 L of absolute alcohol consumed per year, is the second highest in the world [2]. This has serious consequences for public health, as the risk for most disease and injury increases with rising levels of consumption [19,20]. Thirdly, how alcohol is consumed in a country is an important determinant of types and levels of problems associated with drinking. According to WHO, the most common pattern of drinking in Africa is heavy episodic use, defined as consumption of 60 or more grams of pure alcohol (6+ standard drinks in most countries) on at least one single occasion at least monthly, which has a high potential for causing health or social harm [2,7,21].

Alcohol consumption has been identified as the leading risk factor for death and disability in sub-Saharan Africa and the leading risk factor for disability-adjusted life-years (DALYs) among African male adolescents aged 15–24 years [22]. In a recent publication, Ferreira-Borges and colleagues [5] concluded that alcohol was responsible, in 2012, for 6.4% of all deaths and 4.7% of all DALYs in the African Region. These figures present a higher number of deaths and DALYs attributable to alcohol in the region than that previously estimated by Roerecke et al. [21] for 2002 (2.2% of all deaths and 2.5% of all DALYs) and by WHO for 2012 (3.3% of all deaths and 2.4% DALYs) [2]. They basically reflect the impact of alcohol-consumption on the incidence and course of HIV/AIDS. These results are consistent with previous evidence showing that alcohol consumption and especially heavier dinking patterns are linked with many facets of HIV disease, such as unsafe sex [23,24,25], reduced adherence to antiretroviral treatment (ART) [26], immune system impairment and drug interactions and hepatotoxicity [27]. This burden of mortality is non-trivial (and even higher than the global alcohol-attributable death rates), and demands a stronger response from countries to address the consequences of excessive alcohol consumption. 

## 3. Evolution of Countries’ Response

In most countries in Africa a general perception persists that there are more urgent public health problems than harmful use of alcohol. Because infectious diseases still outnumber chronic diseases as a cause of death in most countries and because the link between alcohol use and HIV and tuberculosis is poorly understood, alcohol-related harm continues to be neglected [28]. This position is reinforced by international donors and their funding priorities. According to a WHO Bulletin paper, half of the funding provided by large donors between 2000 and 2009, called development assistance for health, targeted only two diseases: HIV and malaria [29,30].

Recognition of the impact of alcohol on health and on the need to develop adequate policy responses began to change in 2007 with the technical support provided by WHO and the active leadership from some countries at the global level in important global health initiatives. In 2008, Kenya and Rwanda took an active role at the World Health Assembly proposing a draft resolution that would lead to the adoption of the “Global strategy to reduce harmful use of alcohol” [31]). In 2010, Ministers of Health adopted the “Regional Strategy to reduce harmful use of alcohol in the African Region” [15]. In addition, in 2011, countries committed themselves to reduce alcohol-related harm, among other risk factors for NCDs, at the United Nations General Assembly [32] and endorsed in 2013, the WHO Global Action Plan for the Prevention and Control of Non-Communicable Diseases (NCDs) [16] where they set a target of at least a 10% relative reduction in harmful use of alcohol. In 2016, at the World Health Assembly, African countries requested to set up a thinking group to look at harmful use of alcohol as a factor for NCDs [33]. 

Despite these international developments, the implementation of evidence-based solutions to minimize the harmful use of alcohol in the continent has been insufficient. In 2008/2009, countries collaborated in the WHO Global Survey on Alcohol and Health. This process showed that out of the 46 countries in the Region, only 10 countries had recently implemented alcohol policies and that coordination with relevant sectors and within government was lacking. It also showed that appropriate skills, knowledge, and resources for the scaling up of adequate policy measures were missing [2]. A recent study by Ferreira-Borges and colleagues [34] also found substantial room for policy improvement across the majority of the continent in areas such as availability (where countries earned only 36% of the points possible) and drink-driving policies (where countries gained only 41% of the points possible). Unfortunately, the WHO Framework for Implementing the Regional Strategy to Reduce the Harmful Use of Alcohol (2014–2020), which set important indicators to monitor the implementation of the Regional Strategy to reduce harmful consumption, was never implemented.

## 4. Barriers and Way Forward

The reasons for insufficient implementation of evidence-based policies in Africa are linked to specific barriers, some of which have been increasing in recent years. First, the lack of information about the nature and extent of harmful use of alcohol, especially in an environment of unrestrained marketing and other industry activities, weakens the ability of policymakers to take appropriate policy measures, namely those linked to alcohol availability [35]. With much of society being unable to recognize the harmful effects of alcohol consumption and the scope of the alcohol industry’s strategies to normalize regular drinking and attract new consumers, alcohol control efforts aimed at regulating availability are likely to be misunderstood or be seen as ineffective. Adding to the cultural importance given to alcohol in social engagements and traditional rituals like marriage ceremonies, kingship enthronements, religious rituals, and even funerals [7,36], intensive and unregulated alcohol marketing strategies in many countries across Africa are contributing to shape the social environment in which the positive aspects of drinking are dominant. Drinking is portrayed as an emblem of success, and a symbol of heroism, courage and virility, all ingredients that contribute to an unhealthy drinking culture [6,37,38,39,40,41,42]. Although alcoholic beverage advertisements and other marketing materials are subject to restrictions in many countries, in most countries in Africa, alcohol industry groups are using self-regulation guidelines as the sole means to protect vulnerable populations. This comes as a consequence of the almost total absence of binding regulations on alcohol advertising, including sponsorship and sales promotion in most African countries [2,43]. The industry’s self-regulation codes describe which types of content (and exposure markets) the industry will voluntarily exclude [44]. However, several studies in African countries have shown that the alcohol industry’s self-regulation scheme is systematically violated and therefore does not protect young people from exposure to potentially harmful alcohol marketing [38,45]. Moreover, a recent systematic review of studies investigating the content of, and exposure to, alcohol marketing in relation to self-regulated guidelines has shown that alcohol advertisements consistently violate the content guidelines of alcohol marketing self-regulatory codes and contain themes that could be considered inappropriate for children, adolescents and other vulnerable populations [46]. There is general consensus among public health experts that the most effective response to alcohol marketing in the African region would be a comprehensive ban on alcohol advertising, promotion and sponsorship [47,48], with exceptions at points of sale. However, if that is not possible, the French Loi Evin model [49], which specifies within reasonable commercial boundaries the marketing activities that can be safely permitted, could be used as an alternative approach. Also very important would be the development by WHO of a common code for alcohol marketing regulation and practice, at the global level, to support countries’ policy efforts in situations where a complete ban is not feasible, at least in the short term [47].

Secondly, a big part of the alcohol industry strategy has been to limit the statutory regulation of alcohol, and to steer government policy into protecting its own interests. This has initially been done by developing close ties with government policymakers by offering help to develop alcohol-related policies [43]. However subsequent to the publication of an analysis showing SABMiller’s direct involvement in writing industry-favorable policy documents [43], the alcohol industry has shifted to more subtle investments in public relations, active lobbying and promotion of “corporate social responsibility” (CSR) as a business strategy [50,51]. Philanthropic sponsorships or entrepreneurship development programmes targeting youth allow global alcohol producers to represent themselves as “responsible” corporate citizens and consequently influence decision makers and public opinion [50,52,53]. The alcohol industry has also become very active in blocking any policies that might constitute a possible threat to their business interests [54]. This has been the case with policy development in Malawi, which was delayed for more than 7 years before it could be implemented [55]. Similar tactics were used in Botswana, involving high-profile legal battles [56] and recently in South Africa with the proposal for a marketing ban [37,57] and with the 2016 Liquor Amendment Bill which aims among other things to raise the drinking age in South Africa [58,59]. The industry’s resistance to effective alcohol policy, including various kinds of financial contributions at all levels of government [60], and to members of other parties, deserve further in-depth investigation. To overcome these obstacles, governments need to make clear that the alcohol industry has no role in the formulation of alcohol policies [57,61], and partnerships with the commercial alcohol industry, its “social aspects organizations” and other groups substantially funded by the commercial alcohol industry should be avoided. For policymakers and those directly implicated in policy formulation, there should be a rigorous adherence to Conflict of Interest principles [60] to ensure that the integrity of official decision-making is not compromised by public officials’ private interests. Unfortunately, some regimes in Africa are highly dependent on the breweries and this makes it impossible to separate policy decisions from industry business. For example, in Burundi, Heineken operates in a joint venture with the government and completely dominates the national economy. It represents 10 percent of the national income and 30 percent of all tax revenues [62].

Thirdly, unrecorded consumption and its “fatal consequences” are being used by the alcohol industry to prompt some governments to rethink the prohibition of traditional home brewed alcohol and allow for tax breaks for industrialized production of alcoholic beverages aimed at the lower end of the market [10,11]. However, there is currently no scientific evidence for the conclusion that much of the non-commercial alcohol is contaminated or toxic [63]. Researchers suggest that given current knowledge, the most important current threat of unrecorded alcohol may stem from an associated heavy drinking consumption pattern combined with high alcohol strength in beverages and not from additional harmful components in those drinks. From a public health perspective, the industry strategy to develop inexpensive commercial brands to compete with the informal market increases the risk of cheap commercial alternatives and a massive increase in formal production. With the increase in production, availability of alcoholic beverages to the general population will also be increased [64,65]. The alcohol industry’s global strategy to increase alcohol consumption in Africa needs to be carefully considered and its impact studied. Solutions will also need to involve other sectors besides health.

Fourthly, while pricing and taxation policies have the most evidence of effectiveness [66,67,68,69], these measures have not yet been fully explored in the continent in terms of their public health and revenue generation benefits. As explained above, the industry is using unrecorded alcohol as a strategy to justify tax reductions, and several countries like Uganda and Mozambique have already reduced taxes on alcohol [10]. Botswana was one of two countries in Africa with substantial alcohol tax increases; however, the funds derived from the alcohol levy in this country have gone into the general fiscus, youth development initiatives with little proven outcome, and into alcohol awareness programmes that have no evidence of effectiveness. The other country was Nigeria that had a 60% increase in alcohol taxes in 2016 [70]. From the experiences in the tobacco field, the amount collected with additional taxes should be allocated, totally or in part, to an independent, autonomous structure (i.e., a foundation) free from political influence and bureaucratic interference [71,72]. From our perspective, harmful use of alcohol in African countries cannot be reduced unless there is an increase in the proportion of alcohol that is taxed and there also should be increases in the unit price of alcohol. These are achieved by raising taxes and better enforcement of tax regimes. However, owing to the size of the home-brew market, strategies should be comprehensive in order to address the complex web of traditional and home-brewed alcohol and also include other non-price interventions to reduce harmful use of alcohol.

Fifthly, to reduce availability in many African countries, important efforts are needed to bring unlicensed outlets, like shebeens, where traditional, home-brewed alcoholic beverages are produced, into the regulated market. However issues linked to bribery, lack of human and financial resources and the role of shebeens in employment, especially for women, have prevented attempts to address this issue [73,74,75,76,77]. In our view, community information and mobilization, and support mechanisms for alternative means to generate income need to be put into place before adopting license-enforced restrictions. Without sufficient popular support, enforcement and maintenance of any restriction is more difficult and resistance and circumvention can develop. Some of these ideas are now contained in the Western Cape (South Africa) Alcohol Harms Reduction Green Paper that was put out for public comment in the last quarter of 2016 [78,79].

Sixthly, although human and financial resources may be an important barrier, countries have been missing opportunities to implement important responses in primary health care and at the community level. Despite mounting evidence, many countries with heavy NCD related diseases and HIV/TB and alcohol burdens do not fully recognize these epidemics as intrinsically interrelated [27,80]. As a consequence, alcohol is not integrated in NCD and HIV/TB interventions, resulting not only in missing opportunities for improving health of the population within existing facilities in countries but also in reduced efficacy of the interventions provided [81]. The potential value of introducing alcohol-related preventive measures in primary health care and specialised HIV clinics, aimed at reducing alcohol consumption among people with HIV/AIDS or TB has already been noted [80,82,83]. Better links need to be made between these agendas and alcohol. Targeted interventions are needed in high risk venues and aimed at high risk populations (e.g., persons frequenting shebeens/bars, servers/managers/owners, or at PHC level in patients with NCDs). People living with HIV/AIDS and TB need better information on how alcohol affects their immune system and interacts with ARVs and other medications, and alcohol’s link with sexual risk behavior and with medication compliance [80].

A final barrier that countries in Africa will need to address is linked to the threats imposed by global trade treaties. These treaties, aimed at reducing barriers, increasing competition, lowering prices and promoting consumption, are the opposite of what alcohol control measures aim to achieve [84]. International trade agreements involving alcohol products could undermine whatever governments do at a national level in terms of improving population health [85]. Therefore, governments need to recognize the inherent conflicts between free trade and public health and to work to exclude alcohol and tobacco from trade agreements. Accepting that the alcohol trade is not a wholly positive force in the economy, nor a platform to increase national sustainable development, and that the net economic impact of alcohol on a country is likely to be negative, is an important step for public health in the context of this debate. 

Cross-cutting actions like increasing health information to the public (such as health, economic and social impact analyses and evaluations of industry tactics) and empowering public health activists (professionals, scientific organizations and NGOs) to advocate for public health and argue for effective policies can help reduce some of these barriers. More resources are needed to mobilize civil society groups. Unfortunately, except for the members of the Eastern and Southern African Alcohol Policy Alliances, there are no visible groups of NGOs working on alcohol control in Africa. Furthermore, research is undoubtedly needed on the factors that prevent countries from establishing clear alcohol control policies and on the role of evidence in alcohol policy decision making. Country specific case studies would help to understand contextual barriers for policy implementation, including interactions of competing interests’ groups. 

## 5. Alcohol Industry: An Expansive Trajectory in Africa and the Need for Appropriate Regulation

Alcohol industry involvement and investment is rising throughout the African continent, following a general strategy to increase demand, availability and access to alcoholic beverages [11]. In April 2016, the Financial Times reported on a 6% increase in alcoholic beverages volume from January through March and a 12% boost in revenue for SABMiller in Africa [86]. The alcohol industry in Africa is now dominated by large multi-national corporations whose size and profitability help to finance marketing on a global scale [10]. Size also allows resources to be devoted to promoting the policy interests of the industry to create and maintain an environment favorable to its economic and political interests. Industry efforts to shape beliefs about alcohol consumption and to improve the image of international alcohol corporations are increasing throughout the continent. 

The heavy competition among larger beer producers, such as Heineken, SABMiller and Diageo has prompted analysis of where companies could most easily expand their share of the market [10], and new strategies to increase market share have been developed targeting specific populations, such and young people and women [10,11,87,88,89]. Flavoured alcoholic beverages and cider are fast growing categories together with wine, brown spirits and of course, beer. For Anheuser-Busch InBev (AB InBev), Africa’s share of the world’s beer volume is expected to nearly double to 8.1% between 2000 and 2025. In Nigeria, Guiness’s hugely popular Orijin brand, with a higher alcohol content (6%), now has a market share of 50% in its sector since it entered the market in 2013 [4]. At the low end of the market, SABMiller developed inexpensive malt beverages made from local products in order to target those with lower incomes, while managing to obtain tax breaks from some governments (for replacing homebrewed consumption). In a continent with increased economic growth, a young population and the potential for converting traditional abstainers, especially females, to regular drinkers, the alcohol industry is expecting a major growth in its profits. Projections of growth at almost three times the global rate [90] have led to the merger of two alcohol giants: SABMiller, the established industry leader in the continent and AB InBev. This expansive trajectory threatens global health [91] and, in particular, health in Africa [14]. As pointed out by Hanefeld and colleagues [14], market entrance and corporate consolidation of this type leads to increases in consumption, usually implemented through predatory pricing allied with sophisticated branding. 

The certainty that Africa will be a critical driver for the future growth of the global alcohol industry has led AB InBev to accept the European Commission conditions which request the sale of almost all holdings of SABMiller in Europe. This agreement shows how much AB InBev is willing to give up in order to capture other markets, namely Africa [86]. Before merging, AB InBev launched four “Smart Drinking Goals” (Global SDGs), an USD $1 billion initiative to reduce the harmful use of alcohol, while maintaining the traditional argument that the aim is merely to “empower consumers through choice” [92]. This strategy opportunistically uses the Sustainable Development Goals (SDGs) as the rationale for collaboration. Anderson and Rehm [93] make a very useful proposal regarding the evaluation of this initiative that would allow public health advocates and policymakers to use current research capabilities to evaluate the impact, positive or negative, of AB InBev on reducing the harmful use of alcohol and subsequent public health. Given what is known about effective alcohol policy and the nature of industry initiatives, we believe that “Smart Drinking Goals” is unlikely to succeed, not only because an independent and credible steering committee will not be accepted, but also because the expansive trajectory of this alcohol giant has Africa as a critical driver for the growth of its business.

Even if the industry succeeds in promoting low alcohol beers, this will not change the trajectory of increased consumption and with it, increased alcohol-related harm. Changing this trajectory will require innovative leadership from governments and from international agencies charged with protecting health, children’s rights and development. Without it, Africa is likely to face a future involving an ever increasing burden of death and disability associated with alcohol use.

## 6. Conclusions

Strategic shifts in particular policy areas, such as taxation, marketing and controls on availability are needed to reduce harmful use of alcohol in sub-Saharan Africa. This includes taking bold steps to: (1) make sure prices are high (but not so high as to drive consumers into the informal market); (2) ban alcohol advertising except at points of sale; (3) reduce alcohol availability (e.g., by regulating hours/days of sale, sale to minors, and high density outlets); (4) implement effective, evidence-based drink driving countermeasures; (5) use existing opportunities to integrate alcohol education and treatment into NCD and HIV/TB approaches; (6) ensure that international trade treaties do not undermine countries’ efforts to control harmful use of alcohol; and (7) increase research funding in this area. All of these measures require strong political commitment and adjustment of policy responses to protect the health of the African population.
